# EMP2 promotes hepatocellular carcinoma proliferation and invasion by activating cellular autophagy

**DOI:** 10.32604/or.2024.043948

**Published:** 2025-01-16

**Authors:** HAIYING PANG, FENGBO WU, YU ZHANG, NAN ZHANG, CHUNTING WANG, QIU LI, GU HE, PENG ZHANG

**Affiliations:** 1 Department of Pharmacy, State Key Laboratory of Biotherapy, West China Hospital, Sichuan University, Chengdu, 610041, China; 2 State Key Laboratory of Southwestern Chinese Medicine Resources, College of Medical Technology and School of Pharmacy, Chengdu University of Traditional Chinese Medicine, Chengdu, 611137, China; 3 Department of Radiation Oncology, Radiation Oncology Key Laboratory of Sichuan Province, Sichuan Clinical Research Center for Cancer, Sichuan Cancer Hospital & Institute, Sichuan Cancer Center, Affliated Cancer Hospital of University of Electronic Science and Technology of China, Chengdu, 610041, China

**Keywords:** Epithelial membrane protein 2, Hepatocellular carcinoma, Autophagy, Integrin pathway

## Abstract

**Background:**

EMP2 is a tumor-associated membrane protein belonging to the GAS-3/PMP22 gene family. EMP2 expression demonstrates significant tissue specificity and heterogeneity in various human tissues and tumor tissues, where it may play a role in either promoting or inhibiting tumor growth. This study aimed to investigate the expression level, biological functions, and molecular mechanisms of EMP2 in liver cancer.

**Methods:**

we analyzed the mRNA expression levels of EMPs family genes in hepatocellular carcinoma (HCC) tissues and normal liver tissues based on the TCGA database and immunohistochemical analysis of tissue microarrays. Subsequently, we constructed HCC cell lines with either knockdown or overexpression of EMP2 to examine the biological functions and molecular mechanisms of EMP2 in tumorigenesis *in vivo* and *in vitro*.

**Results:**

Bioinformatic and immunohistochemical analysis of tissue microarrays have confirmed the significant upregulation of EMP2 in HCC tissues. *In vitro* and *in vivo* studies have shown that downregulation of EMP2 results in a moderate reduction in the proliferation and invasive capacity of HCC cells. Conversely, overexpression of EMP2 enhances the invasive capacity of HCC cells and induces autophagy. Initial investigations into the molecular mechanisms underlying EMP2-mediated enhancement of HCC cell invasion have revealed the dual regulation of EMP2-induced autophagy and the integrin pathway, which synergistically influence the invasive and metastatic potential of HCC cells.

**Conclusion:**

EMP2 holds promise as a diagnostic marker for HCC metastasis and a potential target for targeted therapy.

## Introduction

Hepatocellular carcinoma (HCC) is the third leading cause of cancer-related deaths worldwide, with particularly high incidence rates in Eastern Asia and Sub-Saharan Africa [1,2]. For early-stage HCC, Surgical resection, local ablation, or liver transplantation are the standard treatment options, resulting in a five-year survival rate of over 70% [2,3]. However, due to the absence of signs and symptoms in early-stage HCC, the disease is frequently diagnosed at an advanced stage [4]. Although tyrosine kinase inhibitors (TKIs), such as sorafenib and lenvatinib, and immune checkpoint inhibitors (ICIs), like atezolizumab and Ipilimumab, have demonstrated efficacy as treatment options for unresectable HCC, the development of resistance to conventional chemotherapy ultimately results in a poor prognosis [4,5]. Thus, elucidating the molecular mechanisms involved in HCC tumorigenesis and development, as well as identifying novel molecular targets for HCC treatment and prognosis, is of paramount importance.

Integrins, a diverse family of cell membrane adhesion receptors, modulate the invasive and migratory properties of cells, thereby promoting tumor invasion, growth, angiogenesis, metastasis, and therapeutic resistance [6–8]. Dysregulation of various integrin signaling pathways has been observed in numerous cancers, including HCC [8,9]. Research has demonstrated that Epithelial membrane protein 2 (EMP2) can regulate integrin expression [10,11]. EMP2, a cell surface protein, is a member of the growth arrest-specific 3/peripheral myelin protein 22 kDa (GAS3/PMP22) gene family, exhibits distinct biochemical and physiological functions [12,13]. For instance, EMP2, which is highly expressed in the endometrium, regulates the expression and localization of α_v_β_3_ integrin, a crucial factor for successful blastocyst implantation [14,15]. Moreover, EMP2 may modulate plasma membrane trafficking activity, cell adhesion, and migration through integrins [10,11]. Furthermore, Wadehra et al. demonstrated that EMP2 facilitates the formation and surface trafficking of lipid rafts with GPI-APs and downregulates caveolin expression, resulting in impaired caveolae formation [16,17]. Caveolae play essential roles in endocytosis, intracellular signaling, and the prevention of oncogenic transformation [18–20]. Accumulating evidence suggests that EMP2 is a promising and intriguing molecular target in various malignancies, potentially improving tumor control and survival [21]. Interestingly, the role of EMP2 varies among different tumors, exhibiting either oncogenic or tumor-suppressing expression [22,23]. EMP2 expression is upregulated in ovarian [24], breast [25], endometrial [26,27], and glioblastoma cancer [28]. Evidence suggests that EMP2 serves as a prognostic indicator in these tumors, as its expression is associated with poor survival and/or advanced disease [25,26,29]. Conversely, in urinary bladder urothelial carcinoma, cutaneous melanoma, nasopharyngeal cancer, and B-cell lymphoma, EMP2 overexpression suppresses the viability and proliferation of cancer cells, functioning as a tumor suppressor [30–33].

Despite the detection of EMP2 transcripts in the liver [13], limited studies have investigated the biological function or molecular mechanisms of EMP2 in HCC to date. The main purpose of current research was to elucidate the preliminary expression pattern, biological function, and molecular mechanism of EMP2 in HCC.

## Materials and Methods

### Bioinformatic analysis

The clinical information and gene expression data (Normalized RPKM, reads per kilobase per million) from TCGA database https://cancergenome.nih.gov/ (The Cancer Genome Atlas, Center for Cancer Genomics, Bethesda, MD, USA) for 374 HCC and 50 adjacent normal tissues were collected. R software (version 3.6.3, https://www.r-project.org/) was applied to transform expression data, and a relevant heat map was drawn based on results with WGCNA (Weighted correlation network analysis, ver. 1.69). The abbreviation of different cancer types: ACC (Adrenocortical carcinoma); BLCA (Bladder Urothelial Carcinoma); BRCA (Breast invasive carcinoma); CESC (Cervical squamous cell carcinoma and endocervical adenocarcinoma); CHOL (Cholangio carcinoma); COAD (Colon adenocarcinoma); DLBC (Lymphoid Neoplasm Diffuse Large B-cell Lymphoma); ESCA (Esophageal carcinoma); GBM (Glioblastoma multiforme); HNSC (Head and Neck squamous cell carcinoma); KICH (Kidney Chromophobe); KIRC (Kidney renal clear cell carcinoma); KIRP (Kidney renal papillary cell carcinoma); LAML (Acute Myeloid Leukemia); LGG (Brain Lower Grade Glioma); LIHC (Liver hepatocellular carcinoma); LUAD (Lung adenocarcinoma); LUSC (Lung squamous cell carcinoma); MESO (Mesothelioma); OV (Ovarian serous cystadenocarcinoma); PAAD (Pancreatic adenocarcinoma); PCPG (Pheochromocytoma and Paraganglioma); PRAD (Prostate adenocarcinoma); READ (Rectum adenocarcinoma); SARC (Sarcoma); SKCM (Skin Cutaneous Melanoma); STAD (Stomach adenocarcinoma); TGCT (Testicular Germ Cell Tumors); THCA (Thyroid carcinoma); THYM (Thymoma); UCEC (Uterine *Corpus* Endometrial Carcinoma); UCS (Uterine Carcinosarcoma); UVM (Uveal Melanoma).

### Regents, cell lines and transfection

The antibodies p-Src (59548S) was purchased from Cell Signaling Technology (Danvers, MA, USA); EMP2 (ab174699) (Accession number: NP_001415), integrin A5 (ab150361) (Accession number: NP_002196), BNIP3 (ab109362) (Accession number: NP_004043), Ki67 (ab16667) (Accession number: NP_002408) came from Abcam (Chicago, IL, USA); LC3 (L7543) (Accession number: NP_852610) was obtained from Sigma (Billerica, MA, USA); SQSTM1 (18420-1-AP) (Accession number: NP_003891), Src (11097-1-AP) (Accession number: NP_005408), Glyceraldehyde-3-phosphate dehydrogenase (GAPDH) (60004-1-Ig) (Accession number: NP_002037) and horseradish peroxidase-conjugated affinipure goat anti-mouse (SA00001-1) and anti-rabbit IgG (SA00001-2), which came from Proteintech (Wuhan, China).

The Bafilomycin A1 (Baf-A1) (S1413) 3-Methyladenine (3-MA) (37979) and ATN-161 (S8454) were purchased from Selleckchem (Houston, TX, USA). Lysotracker (L12492) from Thermo (Carlsbad, CA, USA). The mycoplasma contamination in samples were checked by PCR analysis. The human normal hepatic epithelial cell line THLE-3 and five human hepatocellular carcinoma cell lines, including Huh-7, Hep-3B, HepG2, SNU182, and SK-Hep-1, were purchased from CGMCC (Shanghai, China). THLE-3 were incubated in MEM medium #11095080 (Gibco, Carlsbad, CA, USA) containing 10% fetal bovine serum #A5670701 (FBS, Gibco, Carlsbad, CA, USA), while Huh-7, Hep-3B, HepG2, SNU182, and SK-Hep-1 were seeded in DMEM medium #SH30243 (Hyclone, Logan, UT, USA) containing 10% FBS. The cells above were cultured at 37°C in a humidified environment with 5% CO_2_.

Plasmids for knockdown and overexpression targeting hEMP2, and corresponding empty vector or negative controls were constructed by Vector Builder (Guangzhou, China). For the creation of lentiviral particles, 293 T packaging cells were transiently transfected with the aforementioned plasmids utilizing Lipofectamine 3000 reagent #L3000150 (Thermo, Carlsbad, CA, USA). A 0.45 mm Millipore filter (Millipore, Billerica, MA, USA) was used to collect and filter the viral supernatant, which was then stored in aliquots at −80°C. HepG2 and Huh7 cell lines in logarithmic growth phase were transduced using lentivirus for 3 d, followed by puromycin (2 μg/mL, Invitrogen, Carlsbad, CA, USA) for 3 d to construct hEMP2-stably knockdown cell lines. To obtain hEMP2-overexpressing cell lines, EMP2 overexpression plasmids were combined with Lipofectamine 3000, and added to HepG2 and Huh7 cells. The expression efficiency was determined using qPCR and Western blotting.

### Tissue samples

Six pairs of HCC samples and the corresponding adjacent normal tissues were provided by West China Hospital, Sichuan University. After surgical excision, the tumor tissues were immediately snap frozen in liquid nitrogen and stored at −80°C. The study was conducted in accordance with the Declaration of Helsinki and was approved by the Ethics Committee of West China Hospital of Sichuan University (No. 2022-1986). All patients or family members provided written informed consent.

### Quantitative RT-PCR

Following the manufacturer’s instructions, TRIzol® reagents #9108 (TAKARA, Beijing, China) were applied to extract total RNA from HCC and cell lines. A quantitative amount of RNA was converted to complementary DNA (cDNA) with PrimeScript RT Reagent #RR037A (TAKARA, Beijing, China), followed by qPCR utilizing ChamQ Universal SYBR qPCR Master Mix (#Q711, Vazyme, Nanjing). The relative expression of the target gene expression was calculated with the 2^−ΔΔCT^ formula and the sequences of the primers were listed in Table A1.

### Western blotting

Total protein from homogenized tissues or cell lysates was extracted with a RIPA solution #R0278 (Sigma, Billerica, MA, USA) and Proteasome Inhibitor Cocktail #HY-K0017 (MCE, Monmouth Junction, NJ, USA) and quantified with a BCA kit #23227 (Thermo, Carlsbad, CA, USA). Separation of total proteins by SDS-PAGE (#PG111/PG114, EpiZyme, Shanghai, China) was performed, followed by transferring to PVDF membranes #RIEB02214 (Millipore, Billerica, MA, USA). As part of the blocking process, nonfat milk in phosphate-buffered saline–Tween (PBST) was added to the membranes, and they were then incubated at room temperature for 60 min with matched primary antibodies against cleaved. Membrane incubation with secondary antibodies 1:1000 (ZSGB-BIO, Beijing, China) was performed for 1 h at 37°C after three washings with PBST buffer. The protein bands were visualized by the Touch Imager (e-BLOT, Shanghai, China).

### Cell proliferation assay

The hEMP2-stably knockdown HepG2 and Huh-7 cells in the logarithmic growth phase were added to the 96-well plates with 1 × 10^4^ cells/well. After 0, 1, 2, 3, 4, 5, 6 and 7 days of culture, 100 μL CCK8 reagent #C0038 (Beyotime Biotechnology, Shanghai, China) was added to each well and cultured at 37°C in a humidified incubator with 5% CO_2_ for 0.5–4 h, followed by measurement of OD values (450 nm wavelength) using the Cmaxplus Microplate Reader (Molecular Devices, San Jose, CA, USA). The cell growth curves were plotted with time as the horizontal coordinate and the absorbance value as the vertical coordinate.

### Flow cytometry (FCM) analysis

A flow cytometry assay was performed to detect apoptosis of stable knockdown HepG2 and Huh-7 cells of hEMP2. Briefly, HepG2 and Huh-7 cells in the logarithmic growth phase were placed in six-well plates, and after cell fusion reached approximately 80%, the Annexin V-FITC/PI assay kit #KGA108 (KeyGen Biotech, Nanjing, China) was applied for double staining. Finally, flow cytometry (#2060R, ACEA Biosciences, San Diego, CA, USA) was applied to measure the apoptosis rate. Independent experiments should be repeated at least three times.

### Transwell matrigel invasion assay

To detect the invasive ability of HepG2 and Huh-7 cells with or without hEMP2 knockdown, cells were seeded in the upper layer of transwell chambers (Millipore, Billerica, MA, USA) precoated with Matrigel (#356234, Corning Life Sciences, Corning, NY, USA) and then cultured for 24–48 h at 37°C in a humidified environment with 5% CO_2_. Cells were removed from the upper chamber and fixed with 4% paraformaldehyde for 10 min, followed by crystal violet staining for 20 min. Five random fields were photographed for each transwell using a Axio Imager.Z1 Upright Microscope (Zeiss, Oberkochen, BW, Germany), and the cells in the fields were counted.

### Autophagy assays

The number of autophagosomes of HepG2 and Huh-7 cells knocked down with hEMP2 was calculated by transmission electron microscopy (TEM, Zeiss, Oberkochen, Germany). Briefly, cells were digested with 0.25% trypsin #SM2003 (Millipore, Billerica, MA, USA) until the cells were round and then the cells were fixed in glutaraldehyde for electron microscope. Furthermore, the cells mentioned above were seeded in 96-well plates at a density of 1 × 10^5^ cells/per well and cultured to 70% confluence and transfected with the recombinant GFP-LC3 plasmid (Vector Builder, Guangzhou, China) according to the method mentioned above. After being fixed with 4% paraformaldehyde, cells were then permeabilized with PBST buffer and blocked for 1% with 5% BSA #SW3015 (Solarbio, Beijing, China). After overnight incubation with primary antibody at 4°C, the cells were cultured with HRP-labeled secondary antibody overnight for 60 min at room temperature and then stained with DAPI #10236276001 (Sigma, Billerica, MA, USA). Confocal laser microscope (Olympus, Tokyo, Japan) was administered for image analysis.

### Animal models

All animals were handled according to the animal welfare guidelines and approved by The Ethics Committee of The West China Hospital of Sichuan University (IACUC No. 2021212A). BALB/c nude female mice (6–8 weeks old, weighing 18–20 g) were provided by Beijing Vital River Laboratory Animal Technology Co., Ltd. (China) and reared in the SPF-Class Housing of Laboratory. For the subcutaneous xenograft model, we randomly divided twelve mice into three groups: shEMP2, Vector, and Control (*n* = 4 mice per group). HepG2 cells stably transfected shEMP2 and shNC, and untreated HepG2 cells (4 × 10^6^) were subcutaneously injected into the ribs of mice. The size of the subcutaneous nodules was measured every 3 days. Tumor tissues were isolated from euthanized mice 4 weeks after inoculation. For the lung metastasis model, HepG2 cells stably transfected shEMP2, HepG2 cells stably overexpressing EMP2 and untreated HepG2 cells (2 × 10^6^) were injected into the tail vein of nude mice (*n* = 5 mice per group). After inoculation, nude mice were observed daily for health status, including growth and weight, coat color, and appearance. Tumor tissues were isolated from euthanized mice 6 weeks after inoculation. All tissues including tumor tissues, heart, liver, spleen, lungs, and kidneys of mice, were weighed and fixed with formalin for subsequent examinations.

### H*&*E staining

The tissues of the mice were cut into paraffin sections (4 μm) as previously described. After the tissue sections were dewaxed with xylene, they were rehydrated with an alcohol. Finally, hematoxylin #BSBA-4027 (ZSGB-BIO, Beijing, China) was applied to stain the sections for 5 min, followed by eosin #BSBA-4027 (ZSGB-BIO, Beijing, China) for 3 min.

### Tissue array and immunohistochemistry (IHC)

The human liver tissue array (DC-Liv00009) was obtained from Alenabio, Inc. (Xi’an, China) and analyzed according to the manufacturer’s instructions. In summary, the tissue sections of the human liver tissue array or the mice tumor tissues were deparaffinized and rehydrated and then endogenous tissue peroxides were quenched. The sections incubated with 3% H_2_O_2_ #88597 (Sigma, Billerica, MA, USA) for 10 min and then blocked with 1% BSA for 20 min. The sections were then incubated overnight at 4°C with corresponding primary antibodies (1:1000). Subsequently, the sections were incubated at 37°C for 1 h with HRP-labeled secondary antibodies (1:1000), and then were stained with DAB reagents #D8001 (Sigma, Billerica, MA, USA) and counterstained with hematoxylin. The tissues were examined and imaged with the microscope at 400× magnification. The IHC stains were assessed by figuring an H-score, and H-score greater than 6 indicates high EMP2 protein expression while H-score less than 6 indicates low EMP2 protein expression.

### Statistical analysis

Statistical analysis was evaluated with the GraphPad Prism5.0 software (GraphPad, USA). Data (mean ± SD) were collected from three independent experiments, and differences between experimental groups and control groups were compared with the Student’s *t*-test and one-way ANOVA. Statistical significance was defined as *p*-values less than 0.05.

## Results

### EMP2 is overexpressed in HCC tissues and related to worse prognosis

**Figure 1:**
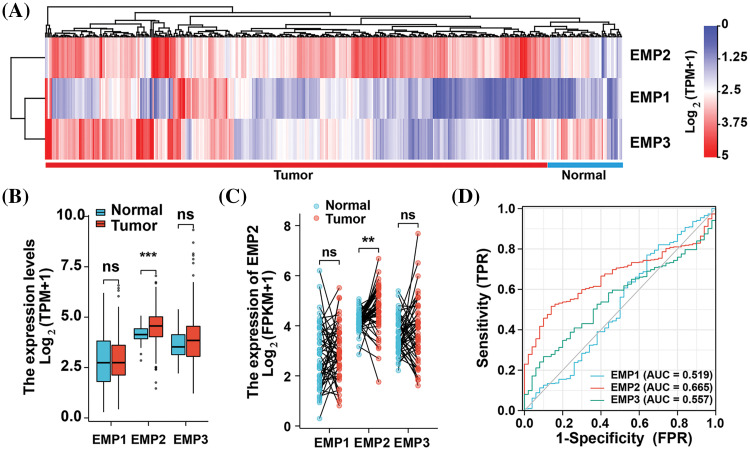
Expression profiles of EMP family proteins in LIHC cohort of TCGA database. (A) Hierarchical clustering analysis of EMPs in liver cancer tissue and adjacent normal tissue. (B) The expression differences of EMP1, EMP2, and EMP3 between tumor and normal samples in the LIHC cohort using the Mann-Whitney U test. (C) Expression differences of EMP1, EMP2, and EMP3 in 50 paired samples. (D) The ROC curves of EMP1, EMP2, and EMP3 in the LIHC cohort using DeLong’s test. ns: not significantly; **p < 0.01; ***p < 0.001.

To investigate the expression patterns of EMP1, EMP2, and EMP3 in hepatocellular carcinoma (HCC) and normal liver tissues, we analyzed mRNA expression data from 374 HCC patients obtained from The TCGA database. EMP1, EMP2, and EMP3 expression was detected in all 374 samples, which included 50 paired samples of HCC tissue and adjacent non-cancerous tissue. The analysis revealed distinct expression patterns for these genes. EMP1 demonstrated high expression in a small proportion of HCC tissues, while exhibiting extremely low levels or no expression in normal liver tissues and most HCC tissues. Conversely, EMP2 exhibited the opposite pattern, with minimal or no expression in normal liver tissues and high expression in most HCC tissues. In contrast, EMP3 displayed a more dispersed expression pattern, with no significant differences between tissue types. EMP3 exhibited high expression in some tumor and normal tissues, while also showing low or no expression in other tumor and normal liver tissues (Fig. 1A). Differential expression analysis of EMP1, EMP2, and EMP3 in the 374 HCC tissues compared to 50 adjacent non-cancerous tissues was performed (Fig. 1B). The average Log_2_ (TPM + 1) expression level (TPM, transcripts per million) of EMP1 in the Normal group was 2.788 ± 1.279, while in the Tumor group, it was 2.858 ± 1.131. Despite the slightly higher expression levels in the Tumor group compared to the Normal group, the difference was not statistically significant (*p* > 0.05). Similarly, EMP3 exhibited higher expression in the Tumor group compared to the Normal group, but the difference was not statistically significant (*p* > 0.05). In contrast, EMP2 demonstrated significantly higher expression in the Tumor group, with an average Log_2_ (TPM + 1) of 4.516 ± 0.803 compared to 4.165 ± 0.411 in the Normal group (*p* < 0.001). These findings suggest that EMP2 may play a crucial role in liver tissues.

To comprehensively assess the differential expression of EMP1, EMP2, and EMP3 in HCC and normal liver tissues, we performed additional analysis on 50 paired HCC tissues and adjacent non-cancerous tissues to determine their mRNA expression levels (Fig. 1C). The results demonstrated that EMP2 expression levels were significantly higher in the Tumor group compared to the Normal group. The median difference in EMP2 expression between the two groups was 0.518, which was statistically significant (*p* = 0.001). In contrast, EMP1 and EMP3 expression levels in the Tumor group were only marginally higher than those in the Normal group, and these differences were not statistically significant (*p* > 0.05). To evaluate the predictive performance of EMPs expression in HCC, we performed a receiver operating characteristic (ROC) curve analysis and validated the results (Fig. 1D). Regarding the ability to predict HCC, EMP2 demonstrated superior diagnostic efficacy compared to EMP1 and EMP3. These findings suggest that EMP2 has a moderate level of predictive performance, which may have clinical implications in the diagnosis of HCC compared to EMP1 and EMP3. Importantly, EMP2 exhibited significant upregulation in HCC tissues, suggesting its potential role in HCC development.

**Figure 2:**
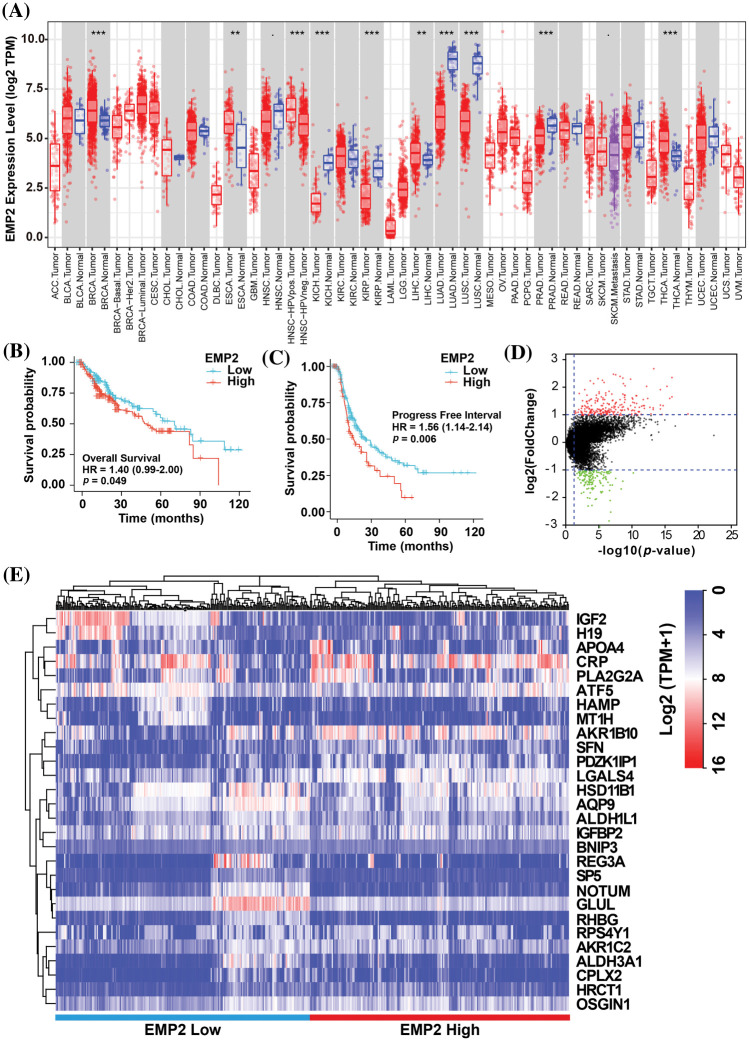
(A) The expression profiles of EMP2 in different cancer subtypes in Oncomine database. And the Kaplan-Meier plots of overall survival; **p < 0.01; ***p < 0.001. (B) or disease-free survival (C) of EMP2 in LIHC cohort. (D) The volcano plot of differential expressed genes between EMP2-high and EMP2-low expressed groups, x-axis represents the log_2_ (fold change) in gene expression, while the y-axis represents -log10(*p*-value). (E) Hierarchical clustering analysis of top ranked autophagy-related genes between EMP2-high and EMP2-low expressed groups.

To investigate EMP2 transcript levels in 36 common human tumors, we analyzed data from the TCGA-TIMER https://cistrome.shinyapps.io/timer/ and Oncomine databases https://www.oncomine.org/resource/login.html. Analysis of the TCGA RNA-seq data revealed significant differences in EMP2 expression levels across various tumor tissues. Among the 18 tumor types with paired normal and tumor samples, EMP2 mRNA expression was significantly higher in BRCA, THCA, ESCA and HCC compared to their respective normal tissues. These results suggest that EMP2 is differentially expressed across multiple tumor tissues. However, the expression pattern is not consistent across all tumor types, with high expression observed in some tumor tissues and low expression in others. This suggests that EMP2 may have distinct roles in the initiation and progression of different cancer types, potentially contributing to the understanding of various malignancies (Fig. 2A). To evaluate the impact of EMP2 expression on overall survival, we performed Kaplan-Meier survival analysis on HCC samples, using the median value of EMP2 mRNA expression in HCC tissues as the cutoff. The results showed that HCC patients with high EMP2 expression had a median survival time of 49.7 months. Conversely, the low-expression group had a median survival time of 70.5 months, with roughly one-quarter of the patients surviving at the 100-month mark (*p* = 0.049, Fig. 2B). Additionally, we performed a comparative analysis of progression-free survival (PFS) in HCC patients (Fig. 2C). The results suggested that EMP2 mRNA expression levels exhibited a significant negative correlation with PFS in HCC patients (*p* = 0.006).

### EMP2 is an autophagy-related gene in HCC

**Figure 3:**
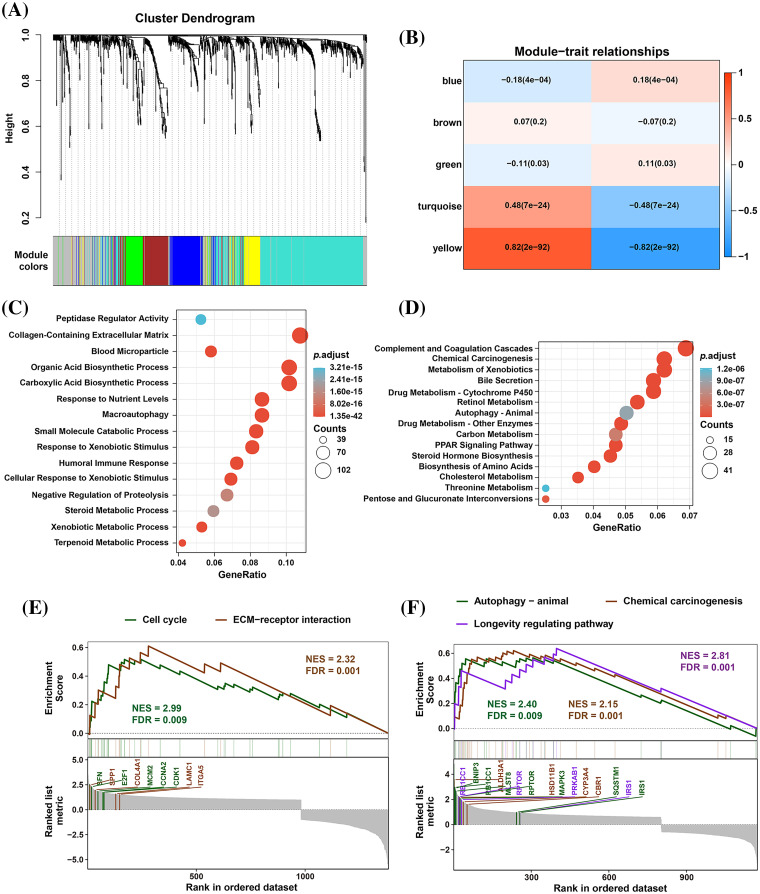
The WGCNA and GSEA analysis of DEGs between EMP2-high and EMP2-low expressed groups. (A) The cluster dendrogram of each module. (B) The correlation efficient of five main WGCNA modules. The enriched GO terms (C) and KEGG pathways (D) of the genes in top ranked WGCNA modules. And the GSEA analysis of these genes by GO terms (E) or KEGG pathways (F).

To identify significantly differentially expressed mRNAs, we performed a differential expression analysis of 374 HCC samples with high or low EMP2 expression. The analysis resulted in the identification of a list of significantly differentially expressed mRNAs. The volcano plot of the differentially expressed genes was generated to visualize the distribution of gene expression profiles (Fig. 2D). Each point on the plot represents a gene, with the color indicating whether the gene is differentially expressed. Furthermore, cluster analysis was performed on the characteristic differentially expressed genes found in the red and green regions, identifying 28 distinct autophagy-related genes. The cluster analysis results are presented in Fig. 2E. Interestingly, some of these genes, including IGFBP2 and BNIP3, are associated with autophagy [34–37]. This finding suggests a potential link between EMP2 and autophagy processes. The WGCNA method was employed to analyze the differentially expressed genes in HCC tissues categorized by high and low expression of EMP2. The hierarchical clustering was initially performed using the hclust function to eliminate outlier sample data. An appropriate soft threshold (b) was calculated as the weighting coefficient for the adjacency function through computation. Subsequently, the WGCNA package was used to compute the correlation matrix and adjacency matrix for EMP2 gene expression profiles, as illustrated in Fig. 3A. Univariate analysis identified two modules, the turquoise module and the yellow module, which exhibited significant associations with high EMP2 expression in HCC tissues (Fig. 3B). GO annotation analysis was further conducted on the 437 genes enriched in the turquoise and yellow modules, revealing key functions such as peptidase regulator activity, collagen-containing extracellular matrix, macroautophagy, small molecule catabolic process, and others (Fig. 3C). Moreover, enrichment patterns of differentially expressed genes were observed in various KEGG pathways, such as complement and coagulation cascades, chemical carcinogenesis, autophagy-animal, carbon metabolism, cholesterol metabolism, and others (Fig. 3D). GSEA pathway enrichment analysis was further performed on these differentially expressed genes. The analysis revealed that these genes were primarily enriched in two GO terms, cell cycle and ECM-receptor interaction (Fig. 3E), and three KEGG pathways, autophagy, chemical carcinogenesis, and longevity regulating pathway (Fig. 3F).

### EMP2 promoted cellular autophagy and invasion in HCC cells

**Figure 4:**
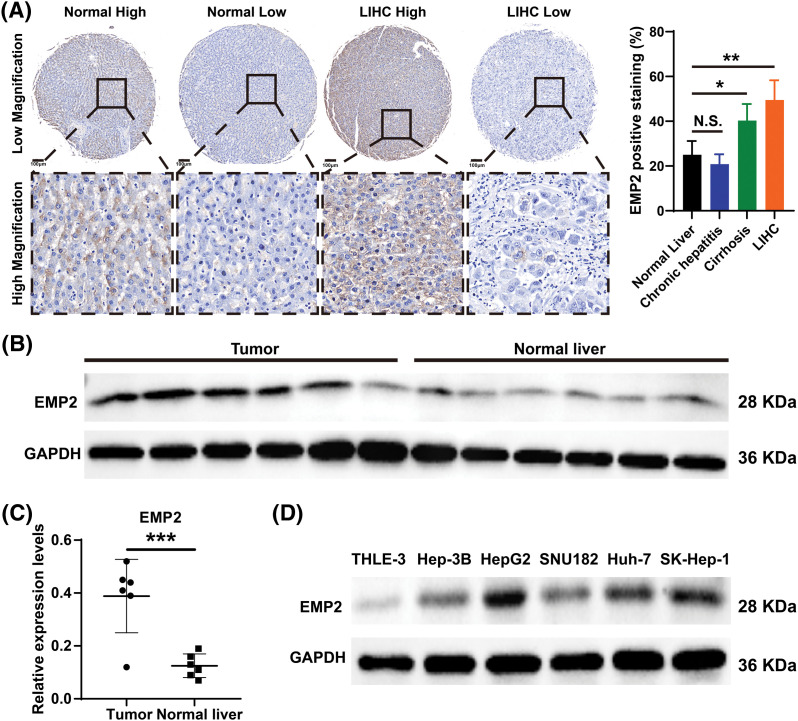
The EMP2 protein expression levels in HCC tissues and cell lines. (A) The representative images of EMP2 IHC staining in HCC and adjacent normal liver tissues, scale bar = 100 μm. (B and C) The western blot of EMP2 in six paired HCC and adjacent normal liver tissues; *p < 0.05; **p < 0.01; ***p < 0.001. (D) The western blot of EMP2 in several HCC cell lines.

**Figure A1:**
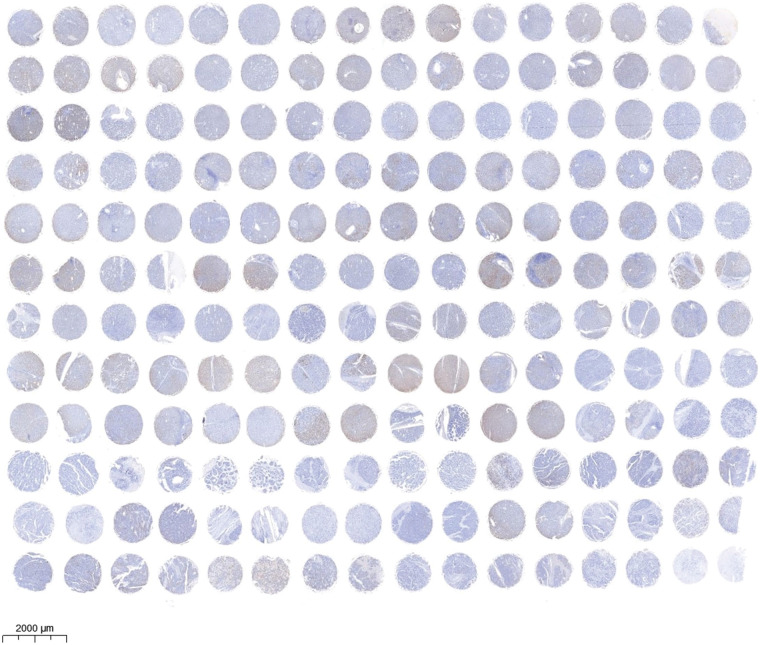
The images of EMP2 IHC staining in the tissue microarray of liver diseases and adjacent normal liver tissues.

Immunohistochemical staining was performed to evaluate the expression level of EMP2 protein in liver disease tissue microarrays. HCC tissue exhibited a significantly higher positive rate of EMP2 compared to normal liver tissue. Furthermore, in HCC tissue, a higher level of EMP2 protein expression was associated with greater morphological heterogeneity and higher malignancy. The EMP2 positive stained ratio were around 20% in the normal liver and chronic hepatitis tissues, and over half of HCC tissue with high EMP2 staining (Figs. 4A and A1, Table A2). EMP2 was primarily localized in the cytoplasm, with minimal nuclear staining observed. The experimental results demonstrated an increasing trend in EMP2 protein expression as liver disease progressed from inflammation to liver cirrhosis and ultimately to HCC. Moreover, the expression level of EMP2 protein in HCC tissue positively correlated with the malignancy grade, indicating a significant role of EMP2 in the progression of liver diseases. To further validate these findings, six pairs of HCC tissues and matched adjacent normal liver tissues were collected. In five out of the six tissue samples, HCC tissues exhibited significantly higher levels of EMP2 expression compared to adjacent liver tissues. However, in one pair of tissues, the difference in EMP2 protein expression between HCC tissues and adjacent liver tissues was not sound (Figs. 4B–4D). In addition, the level of EMP2 protein expression was assessed in various liver or liver cancer cell lines. EMP2 protein expression was low in the normal liver epithelial cell line THLE-3. In contrast, the level of EMP2 protein expression was relatively high in five HCC cell lines (including Hep-3B, HepG2, SNU182, Huh-7, and SK-Hep-1).

**Figure 5:**
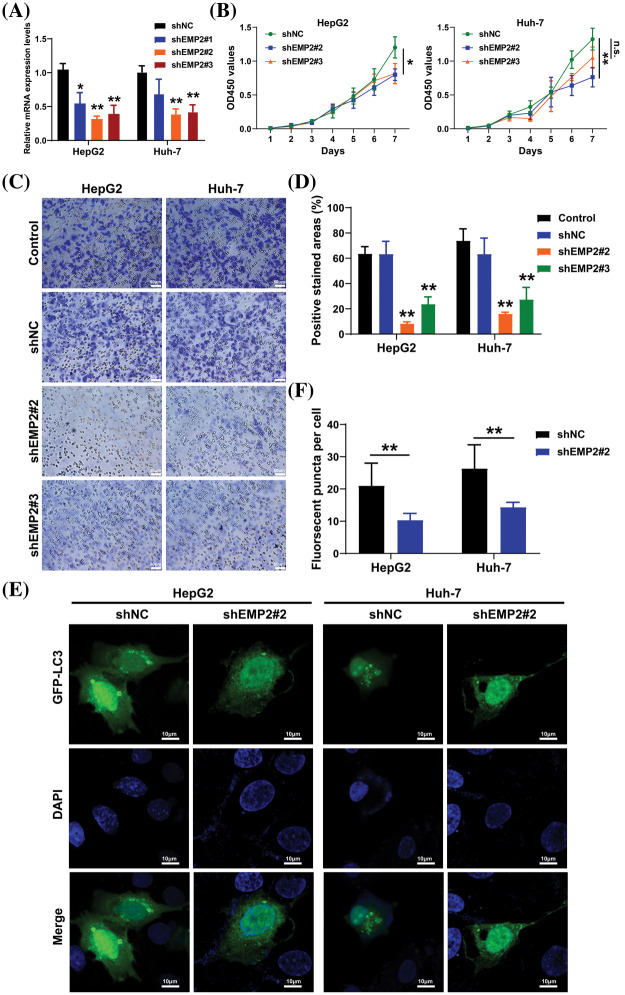
(A) The EMP2 mRNA levels in each group determined by RT-PCR analysis. (B) The cell proliferation curves of HepG2 and Huh-7 cells after EMP2 silencing. (C and D) The cellular invasion capacities of HepG2 and Huh-7 cells after EMP2 silencing, scale bar = 50 μm. (E and F) The GFP-LC3 fluorescent puncta in HepG2 and Huh-7 cells after EMP2 silencing, scale bar = 10 μm; ns: not significantly; *p < 0.05; **p < 0.01.

HepG2 and Huh-7 cells, which exhibited higher endogenous expression of EMP2, were selected, and shRNA-EMP2 lentiviral infection was conducted to screen for stable knockdown of EMP2 in HCC cell lines. The results showed that the three interfering sequences targeting EMP2 effectively reduced the expression of endogenous EMP2 mRNA in HepG2 and Huh-7 cells. In particular, sequences 2# and 3# exhibited relatively high interference efficiency, rendering them suitable for subsequent functional studies (Fig. 5A). Previous findings suggested a close correlation between high EMP2 expression and both clinical staging and prognosis of HCC. Moreover, the proliferative capacity of tumor cells plays a pivotal role in determining their degree of malignancy. To evaluate the impact of stable EMP2 knockdown on HCC cell lines (HepG2 and Huh-7), a CCK-8 assay was performed to measure their proliferation over time. The results showed that the growth curves of HepG2 cells transfected with stable 2# and 3# interference sequences initially overlapped with the control group. However, from day 6 onwards, a noticeable downward trend emerged, suggesting a moderate inhibition of proliferative capacity in the later stages of HepG2 cells (Fig. 5B). Moreover, compared to the blank control (Control) group and the negative control (shNC), stable knockdown of EMP2 expression in HepG2 and Huh-7 HCC cells (shEMP2#2 and shEMP2#3) significantly reduced the migration of tumor cells through the basement membrane and into the lower chamber. This indicates that silencing EMP2 can markedly decrease the invasive ability of both types of HCC cells, with the inhibitory effect being more pronounced for the 2# interference sequence (Figs. 5C, 5D).

**Figure 6:**
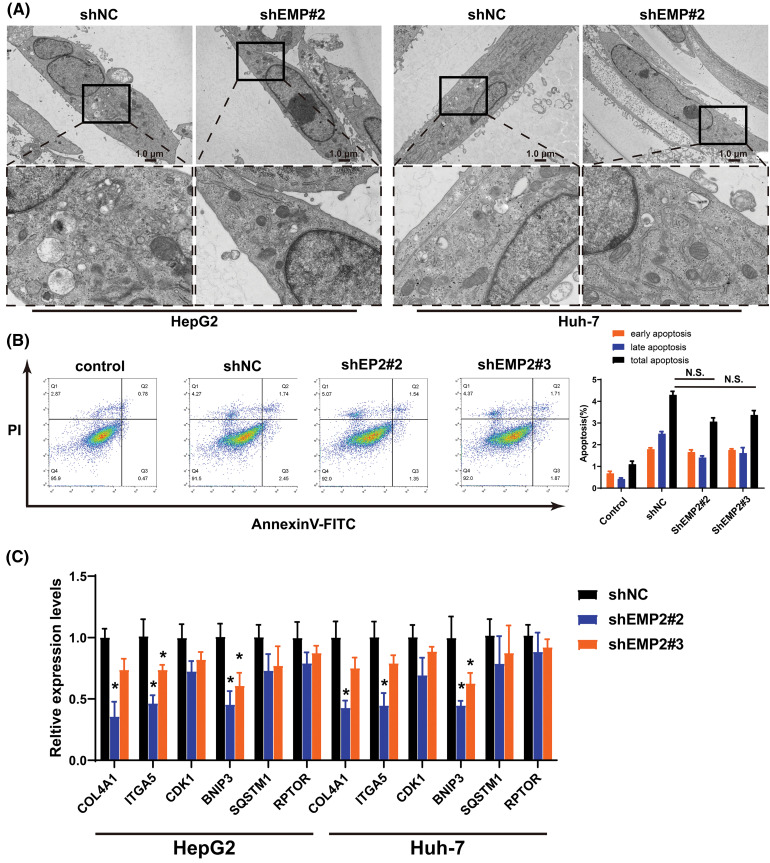

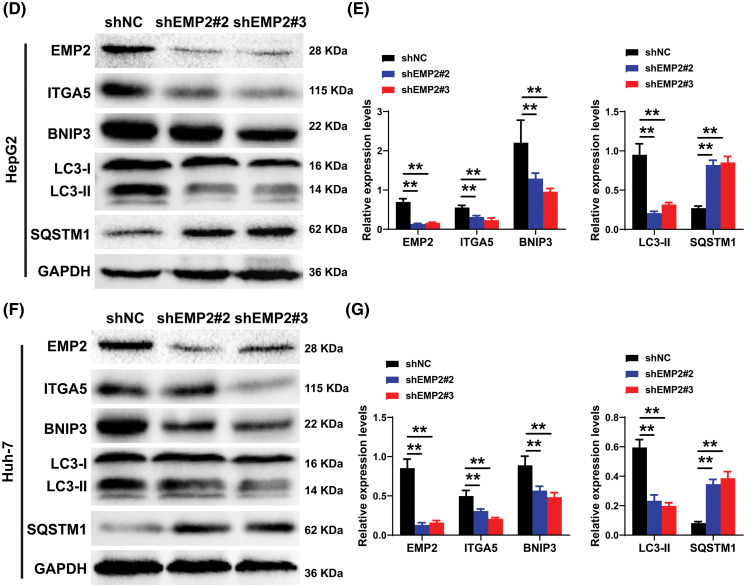
(A) The representative TEM images of HepG2 or Huh-7 cells after EMP2 silencing, scale bar = 1.0 μm. (B) The apoptosis analysis of HepG2 cells by Annexin V/PI dual staining flow-cytometry. (C) The changes on mRNA levels of representative autophagy related genes after EMP2 silencing. And the changes on protein levels of representative autophagy markers after EMP2 silencing in HepG2 (D and E) or Huh-7 cells (F and G); *p < 0.05; **p < 0.01.

Transient transfection of GFP-LC3 (green fluorescent protein-microtubule associated protein 1 light chain 3) plasmids was performed in HepG2 and Huh-7 HCC cells, and the punctate aggregation of GFP-LC3 fluorescence was observed using confocal microscopy. A significant reduction in the number of GFP-LC3 puncta was observed in the cytoplasm of the shEMP2 group compared to the control group, suggesting that downregulation of EMP2 expression in HepG2 and Huh-7 HCC cells substantially inhibits autophagy (Figs. 5E, 5F). To investigate the cellular ultrastructure, TEM was employed to observe cells in each group. The findings demonstrated that in HepG2 and Huh-7 HCC cells, the number of autophagosomes in the shEMP2#2 group cells was significantly reduced compared to the control group, further corroborating the concept that downregulation of EMP2 expression inhibits autophagy in HCC cells (Fig. 6A). Furthermore, Annexin-V/PI (Propidium Iodide) dual-staining flow cytometry was utilized to assess apoptosis in HepG2 cells following EMP2 silencing. The results demonstrated that cellular apoptosis did not exhibit a significant increase in the EMP2 knockdown group compared to the control group of HepG2 cells (Fig. 6B). These findings suggest that although EMP2 silencing inhibited the proliferative capacity and autophagy in HCC cells, it did not trigger cellular apoptosis.

RT-PCR was employed to detect the mRNA expression changes of collagen 4A1 (COL4A1), integrin α5 (ITGA5), Cyclin-Dependent Kinase-1 (CDK1), and autophagy-related genes, including BCL2/adenovirus E1B interacting protein 3 (BNIP3), sequestosome 1 (SQSTM1 or p62), and regulatory-associated protein of mTOR (RPTOR), which are associated with the extracellular microenvironment. The results demonstrated that in HepG2 cells, downregulation of EMP2 expression led to a significant reduction in both ITGA5 and BNIP3 in the shEMP2 group. COL4A1 was significantly downregulated in shEMP2#2, whereas in the shEMP2#3 group, there was a decreasing trend in COL4A1 expression, albeit not reaching statistical significance. CDK1, SQSTM1, and RPTOR exhibited downregulation in both shEMP2#2 and shEMP2#3 groups; however, no statistically significant differences were observed compared to the control group (Fig. 6C). Western blot was utilized to evaluate the expression of several autophagy-related proteins

Figs. 6D, 6E for HepG2 cells and Figs. 6F, 6G for Huh-7 cells. The results revealed that EMP2 protein expression was significantly reduced in the RNA interference group. Concurrently, the levels of LC3-I/LC3-II were significantly decreased, BNIP3 was downregulated, and SQSTM1 protein levels were correspondingly upregulated. These findings further corroborate that significant downregulation of EMP2 inhibits autophagy in HCC cells. Moreover, a substantial downregulation of ITGA5 was observed, suggesting a potential link between EMP2-induced autophagy in HCC cells and integrins.

### EMP2 activated autophagy via integrin signaling pathway

**Figure 7:**
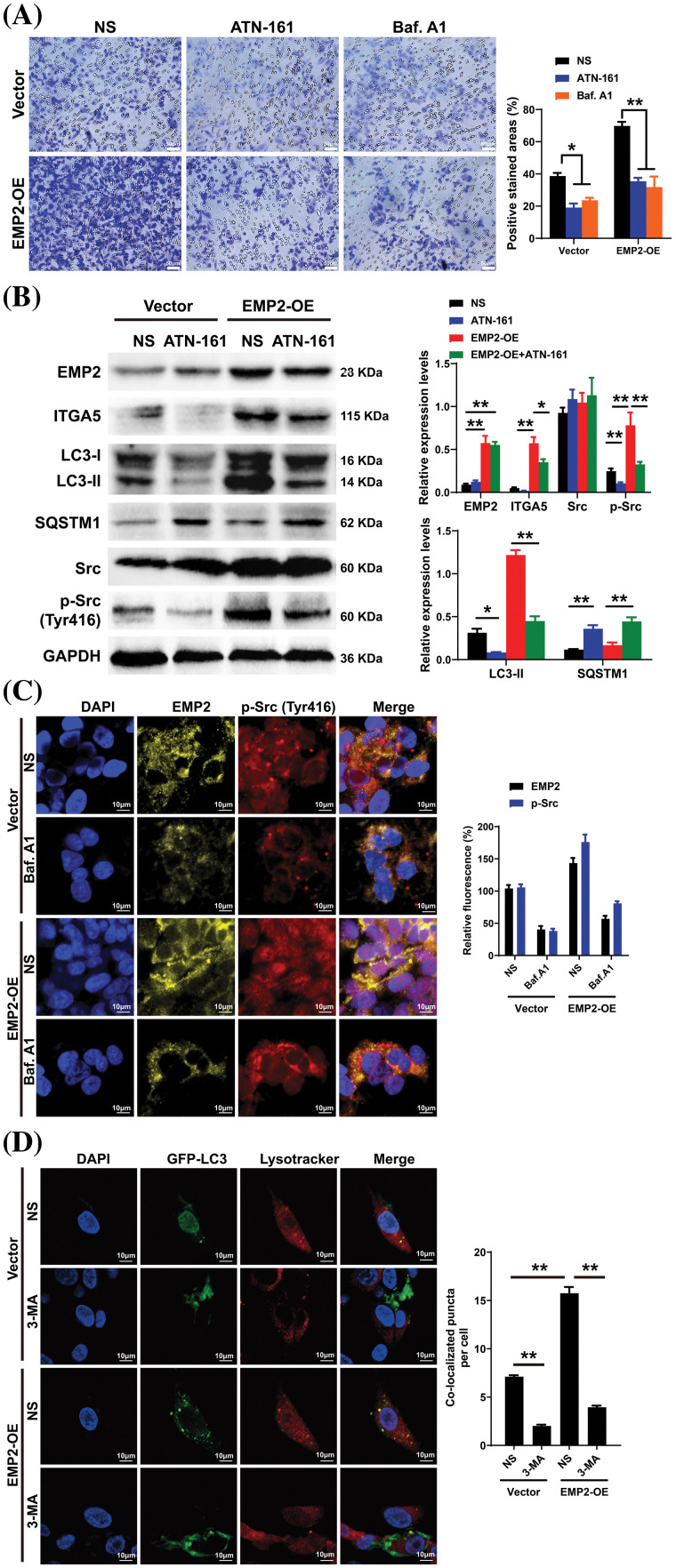
(A) The cellular invasion capacities of HepG2 cells after EMP2 overexpression with integrin inhibitor or autophagy inhibitor (ATN-161: 2.0 μM; Baf. A1: 0.1 μM; 3-MA: 25 μM), scale bar = 50 μm. (B) The changes on protein levels of representative autophagy markers after EMP2 overexpression with integrin inhibitor or autophagy inhibitor. The fluorescent colocalization between EMP2 and p-Src (C) or GFP-LC3 and lysosome (D) after EMP2 overexpression with autophagy inhibitor, scale bar = 10 μm; *p < 0.05; **p < 0.01.

To further elucidate the correlation between EMP2, autophagy, and integrins in HCC, ATN-161, bafilomycin A1 (Baf.A1), and 3-methyladenine (3-MA) were added to HepG2 cells overexpressing EMP2, and the changes in cellular invasion capacities and their effects on related proteins were observed. ATN-161, an integrin inhibitor, has demonstrated anti-tumor efficacy in advanced solid tumors [38]. Baf.A1 and 3-MA are potent inhibitors of autophagy [39,40]. As illustrated in Fig. 7A, both ATN-161 and Baf.A1 significantly inhibit the invasive capacity of HepG2 cells in the extracellular matrix. Furthermore, EMP2 overexpression substantially enhances the invasive ability of HepG2 cells, resulting in a significant increase in the number of transmembrane cells. However, the addition of ATN-161 and Baf.A1 significantly suppresses the promoting effect of EMP2 on the invasive ability of HepG2 cells. These findings indicate that the invasive process regulated by EMP2 is influenced by both integrin and autophagy. Integrins and EMP2 are transmembrane proteins situated on the cell surface [10]. Previous studies have demonstrated a direct interaction between integrins and EMP2, with both being involved in various physiological and biochemical processes, such as cell proliferation and signaling [10,14]. When engaged in regulating cellular processes, integrins can activate multiple signaling molecules, such as Src, recruit kinase complexes, phosphorylate downstream signaling molecules, activate cell signaling pathways, and regulate tumor cell growth and metabolism, thereby influencing tumor development [41,42]. Fig. 7B illustrates that in the empty vector control group, the expression of EMP2 and p-Src is significantly inhibited by ATN-161. In contrast, the addition of ATN-161 suppresses the increased expression of EMP2 and p-Src caused by EMP2 overexpression in the EMP2 overexpression group. Moreover, the evident co-localization of EMP2 and p-Src suggests a direct interaction. These findings indicate that the expression of EMP2 and p-Src, as well as their interaction, are partially regulated by autophagy. As shown in Fig. 7C, the expression of EMP2 and p-Src was significantly inhibited by Baf.A1 in the empty vector control group. However, the addition of Baf.A1 suppresses the increased expression of EMP2 and p-Src caused by EMP2 overexpression in the EMP2 overexpression group (Fig. 7C). Furthermore, EMP2 and p-Src exhibit a certain degree of co-localization, suggesting a possible direct interaction between the two. These results suggest that the expression of EMP2 and p-Src, as well as their interaction, are partially regulated by autophagy. Fig. 7D illustrates that GFP-LC3 was transiently transfected into HepG2 cells of different groups, and the lysosome probe Lysotracker was added to observe the effects of 3-MA and different EMP2 expression states on the interaction between autophagosomes and lysosomes. The results indicate that in the EMP2 overexpression group of HepG2 cells, the number of autophagosomes and the fusion of autophagosomes with lysosomes significantly increased. The enhanced autophagy in HepG2 cells caused by EMP2 overexpression can be partially reversed by 3-MA.

**Figure 8:**
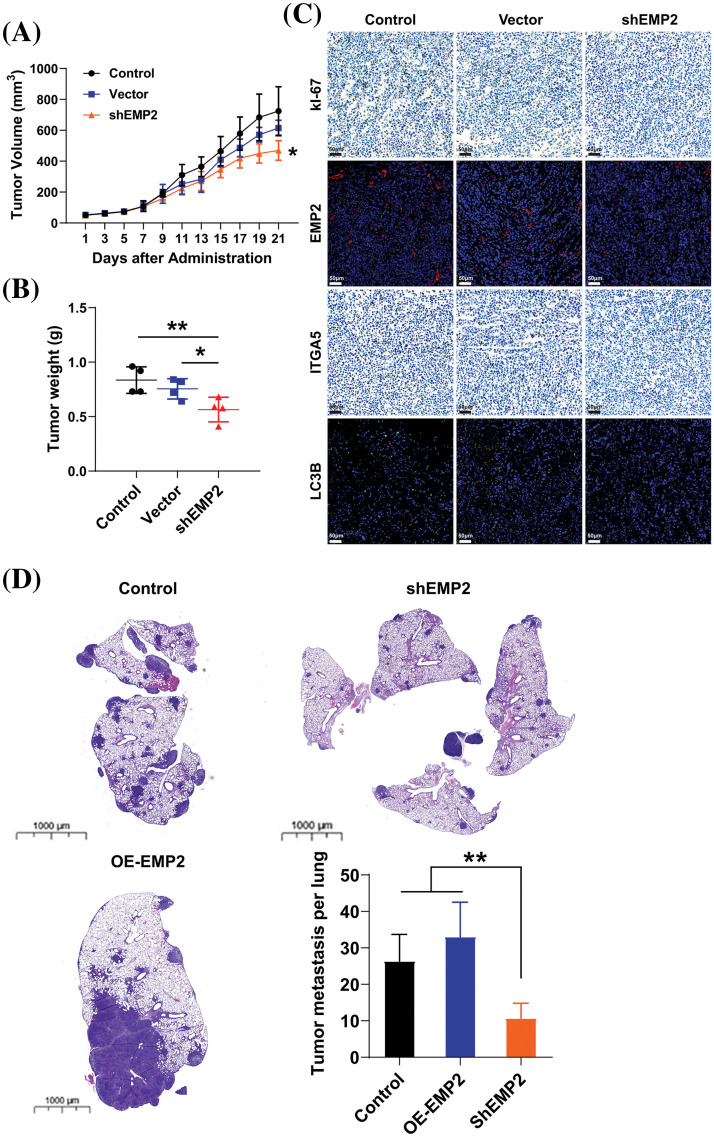
The tumor volume (A) and tumor weight (B) in control or EMP2 knockdown HepG2 cells in the subcutaneous xenograft murine models. (C) The representative IHC or IF images of Ki67, EMP2, ITGA5 and LC3B in each group, scale bar = 50 μm. (D) The representative H&E images of lung tissues in the tumor pulmonary metastasis murine models, scale bar = 1000 μm; *p < 0.05; **p < 0.01.

To further evaluate the role of EMP2 in the growth and proliferation capacity of HCC cells, we established shEMP2 stably transfected HepG2 cells for *in vivo* functional experiments. The results demonstrated a significant inhibition in the growth of HepG2 cells following EMP2 knockdown. Compared to the control group, the EMP2 knockdown group exhibited a significant decrease in tumor growth rate, size, and wet weight (Figs. 8A, 8B). Following 21 days of inoculation, the mice were euthanized, and the intact tumor tissues were embedded and sectioned for immune-histochemical staining of Ki67, EMP2, ITGA5, and LC3B. The results revealed a significant reduction in these markers in the shEMP2 group compared to the control group, indicating a substantial downregulation of EMP2 in the experimental group’s tumor tissues (Fig. 8C). Concurrently, the proliferation capacity of tumor cells decreased, and the expression level of integrin α5 also exhibited a significant decrease. The reduced level of LC3B indicated a decrease in tumor autophagy levels associated with the decrease in EMP2 expression, which is consistent with the *in vitro* results.

## Discussion

Hepatocellular carcinoma, which constitutes over 90% of liver cancers, is one of the most prevalent malignant tumors globally. Its insidious onset and high invasiveness often lead to diagnoses in advanced stages, emphasizing the critical need for research into its pathogenesis, the discovery of new biomarkers, and the development of novel treatments. EMP2’s role in cancer varies markedly across different types; it acts as a tumor suppressor gene in cancers like B-cell lymphoma and melanoma, where it is often downregulated or absent. Conversely, it functions as an oncogene in several other cancers, including glioblastoma and breast cancer, where its upregulation is closely linked to tumor progression and invasiveness. This study is the first to explore EMP2’s expression, biological function, and molecular mechanisms in liver cancer, particularly focusing on the interaction between autophagy in liver cancer cells and EMP2-mediated integrin activation.

**Figure 9:**
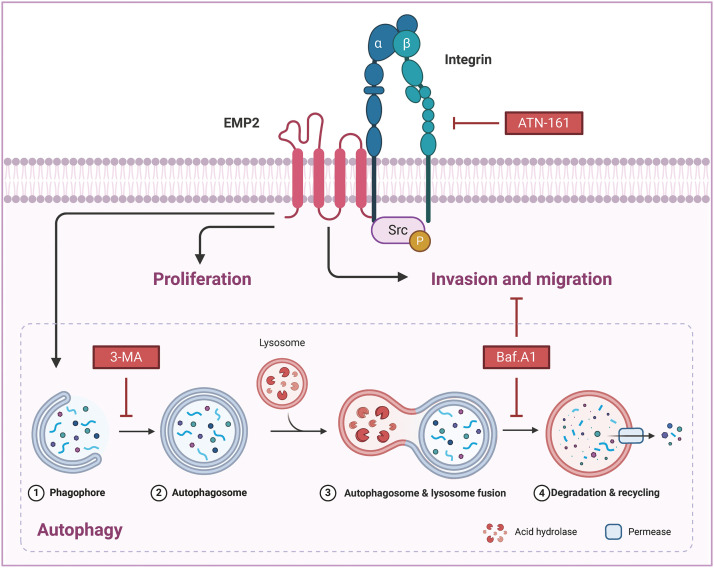
The molecular mechanisms of EMP2 in HCC.

Our analysis of transcriptome data from 374 liver cancer patients from the TCGA database revealed that only EMP2 shows significant differences in expression between liver cancer and normal liver tissues, suggesting its significant role in the onset of liver cancer. EMP2’s expression exhibits organ-specific traits, and numerous studies have reported its varied expression across different tumors. Tissue microarrays indicate that EMP2 levels are significantly lower in low-grade gliomas than in high-grade gliomas; however, in GBM patients, EMP2 expression does not significantly impact survival times. In breast malignant phyllodes tumors, EMP2 is downregulated in epithelial cells but upregulated in stromal components. Moreover, detailed analysis of EMP2 in potential precancerous lesions suggests that EMP2 positivity strongly predicts the progression of endometrial cancer. In nasopharyngeal carcinoma, EMP2 expression is generally weak or absent in tumor tissues but moderate to strong in adjacent non-tumor tissues, with higher levels of EMP2 associated with significantly longer survival times. Our comprehensive analysis of pan-cancer transcriptomic data from databases such as TIMER and Oncomine shows that EMP2 expression varies significantly across different cancers. In BRCA, THCA, ESCA, and LIHC, EMP2 mRNA is significantly elevated compared to normal tissues; meanwhile, in KICH, LUAD, KIRP, PRAD, LUSC, and HNSC, EMP2 mRNA levels are markedly lower than in corresponding normal tissues, confirming previous reports and underscoring the complexity of EMP2’s role in cancer biology. We conducted immunohistochemical analyses to explore the expression patterns of EMP2 protein across various stages of liver disease, ranging from normal liver tissue to hepatocellular carcinoma, using a comprehensive liver disease tissue microarray. Through a comparative analysis of gene expression data from hepatocellular carcinoma samples with high *vs*. low EMP2 expression, we identified 28 genes associated with autophagy, such as IGFBP2 and BNIP3, suggesting a potential linkage between EMP2 and autophagy in liver cancer. Silencing EMP2 markedly reduced the proliferation of HepG2 and Huh-7 cells, particularly in Huh-7, yet did not significantly increase apoptosis in HepG2 cells. EMP2, a protein that spans the cell membrane four times, is crucial for binding various cell surface molecules and facilitates transmembrane transport of materials, influencing cell adhesion and migration—key processes in the metastatic cascade of tumor cells. Therefore, we hypothesize that EMP2 could play a significant role in the invasion and metastasis of liver cancer cells. Further validation came from Transwell assays, which showed that EMP2 knockdown significantly impaired the invasive capabilities of HepG2 and Huh-7 cells through the basement membrane, although scratch tests (Data not shown) indicated no substantial impact on their migration abilities. In liver cancer cells stably transduced with shEMP2 lentivirus, transient transfection with the GFP-LC3 plasmid resulted in a noticeable reduction in the green punctate fluorescence of LC3 in the cytoplasm of the interference group compared to the control group, indicating reduced autophagy. This observation was supported by transmission electron microscopy, which revealed a significant decrease in autophagosomes in the EMP2-silenced cells. Quantitative PCR and Western blot analyses further demonstrated that reducing EMP2 in HepG2 and Huh-7 cells decreased the levels of autophagy-related proteins BNIP3 and LC3 and increased the expression of the protein SQSTM1. Additionally, a significant decrease in the expression of integrin ITGA5 was observed, correlating with the reduced invasive capabilities. These findings collectively confirm that downregulating EMP2 significantly inhibits autophagy in liver cancer cells, impacting their invasive and metastatic potential (Fig. 9).

Integrins, heterodimeric receptors composed of α and β subunits that form non-covalent bonds on the cell surface, mediate interactions between cells and between cells and the extracellular matrix (ECM). They play a crucial role in regulating various cellular physiological processes, including invasion, migration, proliferation, and differentiation. EMP2 and integrins, both being transmembrane proteins, have been reported to interact directly or indirectly through the recruitment of the kinase complex FAK/Src, leading to the phosphorylation of downstream proteins and activation of corresponding signaling pathways, thus regulating the invasive and metastatic behaviors of tumor cells. Our study also found that EMP2 downregulation led to a significant reduction in ITGA5 protein expression, weakening the invasion ability of liver cancer cells and resulting in fewer and smaller experimental lung metastases; conversely, overexpression had the opposite effect. Knocking down EMP2 expression in HepG2 and Huh-7 cells significantly reduced autophagosomes, and the overexpressed EMP2 partially reversed the down-regulated Integrin a5 caused by ATN-161 (Fig. 7B). We also discovered that overexpressing EMP2 could induce autophagy in HepG2 cells, with a significant increase in the autophagy marker proteins LC3-I and LC3-II. Following the addition of the integrin inhibitor ATN-161, EMP2 expression was partially reversed, LC3-II expression significantly downregulated, and SQSTM1 expression significantly upregulated, indicating that the expression of EMP2 and p-Src and their interaction are partially regulated by autophagy. The use of both the integrin inhibitor ATN-161 and the autophagy inhibitor Baf.A1 significantly inhibited the increased invasion ability of HepG2 cells caused by overexpression of EMP2, further validating that the EMP2-promoted invasion process is doubly regulated by integrins and autophagy, highlighting the complex interplay between these molecular mechanisms in liver cancer progression. However, several limitations persist in the current research. Firstly, the study utilized only two HCC cell lines for *in vitro* experiments; incorporating additional cell lines would enhance the generalizability of the findings. Moreover, the interactions between EMP2 and autophagy/integrin pathways require further characterization through multi-omics approaches. The mechanistic insights are predominantly based on correlation; direct and causal evidence is lacking in certain areas. The role of EMP2 in HCC stem cells, drug resistance, and the tumor microenvironment remains uninvestigated. In summary, although novel insights were provided, further robust validation studies are necessary to address these limitations before EMP2 can be developed as a clinical target in HCC.

## Conclusion

Bioinformatics and immunohistochemical analysis consistently demonstrate that EMP2 expression increases as the disease progresses from hepatitis to cirrhosis and ultimately to HCC. Both *in vitro* and *in vivo* studies have proven that reducing EMP2 expression not only inhibits autophagy but also diminishes the proliferation and invasive capabilities of HCC cells. Initial molecular studies indicate that EMP2 enhances the invasiveness and metastatic tendencies of HCC cells by activating integrins and implementing a bidirectional regulatory mechanism, processes that are intricately linked to the autophagy triggered by EMP2. Thus, EMP2 is recognized not only as an effective marker for diagnosing HCC metastasis but also as a potential target for future biotherapeutic interventions against HCC.

## Data Availability

The datasets and raw data in this study are available from the corresponding author on reasonable request.

## Appendix

**Table A1:** The primer sequences for qRT-PCR

Gene name	Forward	Reverse
β-actin (NM_001101)	GGCTCAGCGTCTGGCTG	CACCCTTTTGTCCTGGTGGT
EMP2 (NM_001424)	GCTTCAACTTAGACGCGGAG	ATTCAATGGGGGTGCACTGT
COL4A1 (NM_001845)	CTGGGGTCAGCTCGTTACTC	TCCACTTCTGGCCACACTTC
ITGA5 (NM_002205)	AGGGCTCCTGGGTAGAACT	AGCTACTCCGTCCAGACTCA
CDK1 (NM_001786)	CCGTGAAGGCCTACCTTCTG	TCCTCGTCACTGGAAAAGGC
BNIP3 (NM_004052)	CCCCTACATGCCAGCTGAAC	CTTCCTGCCCCGTGTGATAG

**Table A2:** The clinical information of tissue microarray

Pos.	Age	Sex	Organ	Pathology diagnosis	TNM	Grade	Stage	Type
A1	45	Male	Liver	Normal liver	–	–	–	Normal
A2	45	Male	Liver	Normal liver	–	–	–	Normal
A3	23	Male	Liver	Normal liver	–	–	–	Normal
A4	23	Male	Liver	Normal liver	–	–	–	Normal
A5	27	Female	Liver	Normal liver	–	–	–	Normal
A6	27	Female	Liver	Normal liver	–	–	–	Normal
A7	42	Female	Liver	Normal liver	–	–	–	Normal
A8	42	Female	Liver	Normal liver	–	–	–	Normal
A9	36	Male	Liver	Normal liver	–	–	–	Normal
A10	36	Male	Liver	Normal liver	–	–	–	Normal
A11	21	Female	Liver	Normal liver	–	–	–	Normal
A12	21	Female	Liver	Normal liver	–	–	–	Normal
A13	43	Male	Liver	Normal liver	–	–	–	Normal
A14	43	Male	Liver	Normal liver	–	–	–	Normal
A15	50	Male	Liver	Normal liver	–	–	–	Normal
A16	50	Male	Liver	Normal liver	–	–	–	Normal
B1	16	Male	Liver	Normal liver	–	–	–	Normal
B2	16	Male	Liver	Normal liver	–	–	–	Normal
B3	45	Male	Liver	Normal liver	–	–	–	Normal
B4	45	Male	Liver	Normal liver	–	–	–	Normal
B5	38	Male	Liver	Normal liver	–	–	–	Normal
B6	38	Male	Liver	Normal liver	–	–	–	Normal
B7	40	Male	Liver	Normal liver	–	–	–	Normal
B8	40	Male	Liver	Normal liver	–	–	–	Normal
B9	35	Male	Liver	Normal liver	–	–	–	Normal
B10	35	Male	Liver	Normal liver	–	–	–	Normal
B11	35	Female	Liver	Normal liver	–	–	–	Normal
B12	35	Female	Liver	Normal liver	–	–	–	Normal
B13	23	Male	Liver	Normal liver	–	–	–	Normal
B14	23	Male	Liver	Normal liver	–	–	–	Normal
B15	18	Male	Liver	Normal liver	–	–	–	Normal
B16	18	Male	Liver	Normal liver	–	–	–	Normal
C1	58	Female	Liver	Chronic hepatitis	–	–	–	Inflammation
C2	58	Female	Liver	Chronic hepatitis	–	–	–	Inflammation
C3	60	Male	Liver	Chronic hepatitis	–	–	–	Inflammation
C4	60	Male	Liver	Chronic hepatitis	–	–	–	Inflammation
C5	48	Female	Liver	Chronic hepatitis	–	–	–	Inflammation
C6	48	Female	Liver	Chronic hepatitis	–	–	–	Inflammation
C7	72	Male	Liver	Chronic hepatitis	–	–	–	Inflammation
C8	72	Male	Liver	Chronic hepatitis	–	–	–	Inflammation
C9	52	Male	Liver	Chronic hepatitis	–	–	–	Inflammation
C10	52	Male	Liver	Chronic hepatitis	–	–	–	Inflammation
C11	33	Male	Liver	Chronic hepatitis	–	–	–	Inflammation
C12	33	Male	Liver	Chronic hepatitis	–	–	–	Inflammation
C13	42	Male	Liver	Chronic hepatitis	–	–	–	Inflammation
C14	42	Male	Liver	Chronic hepatitis	–	–	–	Inflammation
C15	56	Female	Liver	Chronic hepatitis	–	–	–	Inflammation
C16	56	Female	Liver	Chronic hepatitis	–	–	–	Inflammation
D1	69	Male	Liver	Chronic hepatitis	–	–	–	Inflammation
D2	69	Male	Liver	Chronic hepatitis	–	–	–	Inflammation
D3	55	Male	Liver	Chronic hepatitis	–	–	–	Inflammation
D4	55	Male	Liver	Chronic hepatitis	–	–	–	Inflammation
D5	49	Male	Liver	Chronic hepatitis	–	–	–	Inflammation
D6	49	Male	Liver	Chronic hepatitis	–	–	–	Inflammation
D7	56	Male	Liver	Chronic hepatitis	–	–	–	Inflammation
D8	56	Male	Liver	Chronic hepatitis	–	–	–	Inflammation
D9	68	Male	Liver	Chronic hepatitis	–	–	–	Inflammation
D10	68	Male	Liver	Chronic hepatitis	–	–	–	Inflammation
D11	56	Female	Liver	Chronic hepatitis	–	–	–	Inflammation
D12	56	Female	Liver	Chronic hepatitis	–	–	–	Inflammation
D13	48	Female	Liver	Chronic hepatitis	–	–	–	Inflammation
D14	48	Female	Liver	Chronic hepatitis	–	–	–	Inflammation
D15	45	Male	Liver	Chronic hepatitis	–	–	–	Inflammation
D16	45	Male	Liver	Chronic hepatitis	–	–	–	Inflammation
E1	62	Female	Liver	Chronic hepatitis	–	–	–	Inflammation
E2	62	Female	Liver	Chronic hepatitis	–	–	–	Inflammation
E3	55	Male	Liver	Chronic hepatitis	–	–	–	Inflammation
E4	55	Male	Liver	Chronic hepatitis	–	–	–	Inflammation
E5	40	Male	Liver	Chronic hepatitis	–	–	–	Inflammation
E6	40	Male	Liver	Chronic hepatitis	–	–	–	Inflammation
E7	55	Female	Liver	Chronic hepatitis	–	–	–	Inflammation
E8	55	Female	Liver	Chronic hepatitis	–	–	–	Inflammation
E9	57	Female	Liver	Chronic hepatitis	–	–	–	Inflammation
E10	57	Female	Liver	Chronic hepatitis	–	–	–	Inflammation
E11	35	Male	Liver	Chronic hepatitis	–	–	–	Inflammation
E12	35	Male	Liver	Chronic hepatitis	–	–	–	Inflammation
E13	67	Male	Liver	Chronic hepatitis	–	–	–	Inflammation
E14	67	Male	Liver	Chronic hepatitis	–	–	–	Inflammation
E15	52	Male	Liver	Chronic hepatitis	–	–	–	Inflammation
E16	52	Male	Liver	Chronic hepatitis	–	–	–	Inflammation
F1	60	Male	Liver	Hepatocirrhosis	–	–	–	Cirrhosis
F2	60	Male	Liver	Hepatocirrhosis	–	–	–	Cirrhosis
F3	52	Male	Liver	Hepatocirrhosis	–	–	–	Cirrhosis
F4	52	Male	Liver	Hepatocirrhosis	–	–	–	Cirrhosis
F5	43	Male	Liver	Hepatocirrhosis	–	–	–	Cirrhosis
F6	43	Male	Liver	Hepatocirrhosis	–	–	–	Cirrhosis
F7	50	Female	Liver	Hepatocirrhosis	–	–	–	Cirrhosis
F8	50	Female	Liver	Hepatocirrhosis	–	–	–	Cirrhosis
F9	25	Male	Liver	Hepatocirrhosis	–	–	–	Cirrhosis
F10	25	Male	Liver	Hepatocirrhosis	–	–	–	Cirrhosis
F11	52	Male	Liver	Hepatocirrhosis	–	–	–	Cirrhosis
F12	52	Male	Liver	Hepatocirrhosis	–	–	–	Cirrhosis
F13	40	Male	Liver	Hepatocirrhosis	–	–	–	Cirrhosis
F14	40	Male	Liver	Hepatocirrhosis	–	–	–	Cirrhosis
F15	48	Male	Liver	Hepatocirrhosis	–	–	–	Cirrhosis
F16	48	Male	Liver	Hepatocirrhosis	–	–	–	Cirrhosis
G1	47	Female	Liver	Hepatocirrhosis	–	–	–	Cirrhosis
G2	47	Female	Liver	Hepatocirrhosis	–	–	–	Cirrhosis
G3	36	Female	Liver	Hepatocirrhosis	–	–	–	Cirrhosis
G4	36	Female	Liver	Hepatocirrhosis	–	–	–	Cirrhosis
G5	35	Female	Liver	Hepatocirrhosis	–	–	–	Cirrhosis
G6	35	Female	Liver	Hepatocirrhosis	–	–	–	Cirrhosis
G7	61	Male	Liver	Hepatocirrhosis	–	*	–	Cirrhosis
G8	61	Male	Liver	Hepatocirrhosis	–	*	–	Cirrhosis
G9	46	Female	Liver	Hepatocirrhosis	–	–	–	Cirrhosis
G10	46	Female	Liver	Hepatocirrhosis	–	–	–	Cirrhosis
G11	67	Male	Liver	Hepatocirrhosis	–	–	–	Cirrhosis
G12	67	Male	Liver	Hepatocirrhosis	–	–	–	Cirrhosis
G13	42	Male	Liver	Hepatocirrhosis	–	–	–	Cirrhosis
G14	42	Male	Liver	Hepatocirrhosis	–	–	–	Cirrhosis
G15	66	Male	Liver	Hepatocirrhosis	–	–	–	Cirrhosis
G16	66	Male	Liver	Hepatocirrhosis	–	–	–	Cirrhosis
H1	67	Male	Liver	Hepatocirrhosis	–	–	–	Cirrhosis
H2	67	Male	Liver	Hepatocirrhosis	–	–	–	Cirrhosis
H3	49	Male	Liver	Hepatocirrhosis	–	–	–	Cirrhosis
H4	49	Male	Liver	Hepatocirrhosis	–	–	–	Cirrhosis
H5	45	Male	Liver	Hepatocirrhosis	–	–	–	Cirrhosis
H6	45	Male	Liver	Hepatocirrhosis	–	–	–	Cirrhosis
H7	38	Male	Liver	Hepatocirrhosis	–	–	–	Cirrhosis
H8	38	Male	Liver	Hepatocirrhosis	–	–	–	Cirrhosis
H9	60	Male	Liver	Hepatocirrhosis	–	–	–	Cirrhosis
H10	60	Male	Liver	Hepatocirrhosis	–	–	–	Cirrhosis
H11	46	Male	Liver	Hepatocirrhosis	–	–	–	Cirrhosis
H12	46	Male	Liver	Hepatocirrhosis	–	–	–	Cirrhosis
H13	54	Male	Liver	Hepatocirrhosis	–	–	–	Cirrhosis
H14	54	Male	Liver	Hepatocirrhosis	–	–	–	Cirrhosis
H15	66	Male	Liver	Hepatocirrhosis	–	–	–	Cirrhosis
H16	66	Male	Liver	Hepatocirrhosis	–	–	–	Cirrhosis
I1	37	Male	Liver	Hepatocirrhosis	–	–	–	Cirrhosis
I2	37	Male	Liver	Hepatocirrhosis	–	–	–	Cirrhosis
I3	46	Male	Liver	Hepatocirrhosis	–	–	–	Cirrhosis
I4	46	Male	Liver	Hepatocirrhosis	–	–	–	Cirrhosis
I5	43	Female	Liver	Hepatocirrhosis	–	–	–	Cirrhosis
I6	43	Female	Liver	Hepatocirrhosis	–	–	–	Cirrhosis
I7	70	Male	Liver	Hepatocirrhosis	–	–	–	Cirrhosis
I8	70	Male	Liver	Hepatocirrhosis	–	–	–	Cirrhosis
I9	28	Male	Liver	Hepatocellular carcinoma	T3N0M0	–	IIIA	Malignant
I10	28	Male	Liver	Hepatocellular carcinoma	T3N0M0	2	IIIA	Malignant
I11	49	Male	Liver	Hepatocellular carcinoma	T2N0M0	2	II	Malignant
I12	49	Male	Liver	Hepatocellular carcinoma	T2N0M0	2	II	Malignant
I13	53	Male	Liver	Hepatocellular carcinoma	T3N0M0	2	IIIA	Malignant
I14	53	Male	Liver	Hepatocellular carcinoma	T3N0M0	2	IIIA	Malignant
I15	55	Male	Liver	Hepatocellular carcinoma	T3N0M0	3	IIIA	Malignant
I16	55	Male	Liver	Hepatocellular carcinoma	T3N0M0	3	IIIA	Malignant
J1	41	Male	Liver	Hepatocellular carcinoma	T2N0M0	1—2	II	Malignant
J2	41	Male	Liver	Hepatocellular carcinoma	T2N0M0	1—2	II	Malignant
J3	52	Male	Liver	Hepatocellular carcinoma	T3N1M0	2	IVA	Malignant
J4	52	Male	Liver	Hepatocellular carcinoma	T3N1M0	2	IVA	Malignant
J5	60	Male	Liver	Hepatocellular carcinoma	T3N0M0	2	IIIA	Malignant
J6	60	Male	Liver	Hepatocellular carcinoma	T3N0M0	2	IIIA	Malignant
J7	50	Female	Liver	Hepatocellular carcinoma	T2N0M0	3	II	Malignant
J8	50	Female	Liver	Hepatocellular carcinoma	T2N0M0	3	II	Malignant
J9	48	Male	Liver	Hepatocellular carcinoma	T2N0M0	2	II	Malignant
J10	48	Male	Liver	Hepatocellular carcinoma	T2N0M0	2	II	Malignant
J11	55	Male	Liver	Hepatocellular carcinoma	T3N0M0	3	IVA	Malignant
J12	55	Male	Liver	Hepatocellular carcinoma	T3N0M0	3	IVA	Malignant
J13	35	Female	Liver	Hepatocellular carcinoma	T2N0M0	3	II	Malignant
J14	35	Female	Liver	Hepatocellular carcinoma	T2N0M0	3	II	Malignant
J15	50	Male	Liver	Hepatocellular carcinoma	T3N0M0	3	IIIA	Malignant
J16	50	Male	Liver	Hepatocellular carcinoma	T3N0M0	3	IIIA	Malignant
K1	46	Male	Liver	Hepatocellular carcinoma	T2N0M0	2	II	Malignant
K2	46	Male	Liver	Hepatocellular carcinoma	T2N0M0	2	II	Malignant
K3	66	Male	Liver	Hepatocellular carcinoma	T3N0M0	3	IIIA	Malignant
K4	66	Male	Liver	Hepatocellular carcinoma	T3N0M0	3	IIIA	Malignant
K5	56	Female	Liver	Hepatocellular carcinoma	T3N0M0	*	IIIA	Malignant
K6	56	Female	Liver	Hepatocellular carcinoma	T3N0M0	*	IIIA	Malignant
K7	38	Male	Liver	Hepatocellular carcinoma	T3N0M0	3	IIIA	Malignant
K8	38	Male	Liver	Hepatocellular carcinoma	T3N0M0	3	IIIA	Malignant
K9	54	Male	Liver	Hepatocellular carcinoma	T3N0M0	3	IIIA	Malignant
K10	54	Male	Liver	Hepatocellular carcinoma	T3N0M0	3	IIIA	Malignant
K11	62	Male	Liver	Hepatocellular carcinoma	T3N0M0	3	IIIA	Malignant
K12	62	Male	Liver	Hepatocellular carcinoma	T3N0M0	3	IIIA	Malignant
K13	58	Male	Liver	Hepatocellular carcinoma	T3N0M0	3	IIIA	Malignant
K14	58	Male	Liver	Hepatocellular carcinoma	T3N0M0	3	IIIA	Malignant
K15	62	Male	Liver	Hepatocellular carcinoma	T2N0M0	3	II	Malignant
K16	62	Male	Liver	Hepatocellular carcinoma	T2N0M0	3	II	Malignant
L1	67	Male	Liver	Hepatocellular carcinoma	T2N0M0	3	II	Malignant
L2	67	Male	Liver	Hepatocellular carcinoma	T2N0M0	3	II	Malignant
L3	62	Male	Liver	Hepatocellular carcinoma	T3N0M0	3	IIIA	Malignant
L4	62	Male	Liver	Hepatocellular carcinoma	T3N0M0	3	IIIA	Malignant
L5	59	Male	Liver	Hepatocellular carcinoma	T3N0M0	3	IIIA	Malignant
L6	59	Male	Liver	Hepatocellular carcinoma	T3N0M0	3	IIIA	Malignant
L7	45	Male	Liver	Hepatocellular carcinoma	T3N0M0	2	IIIA	Malignant
L8	45	Male	Liver	Hepatocellular carcinoma	T3N0M0	2	IIIA	Malignant
L9	32	Male	Liver	Hepatocellular carcinoma	T4N0M0	3	IIIB	Malignant
L10	32	Male	Liver	Hepatocellular carcinoma	T4N0M0	3	IIIB	Malignant
L11	64	Male	Liver	Hepatocellular carcinoma	T3N0M0	3	IVA	Malignant
L12	64	Male	Liver	Hepatocellular carcinoma	T3N0M0	3	IVA	Malignant
L13	52	Male	Liver	Hepatocellular carcinoma	T3N0M0	3	IIIA	Malignant
L14	52	Male	Liver	Hepatocellular carcinoma	T3N0M0	3	IIIA	Malignant
L15	48	Female	Liver	Hepatocellular carcinoma	T2N0M0	2	II	Malignant
L16	48	Female	Liver	Hepatocellular carcinoma	T2N0M0	2	II	Malignant
–	42	Male	Adrenal gland	Pheochromocytoma (tissue marker)	–	–	–	Malignant

Note: *: Hepatocirrhosis with hepatocellular carcinoma.
